# Dynamics of receptor-operated Ca^2+^ currents through TRPC channels controlled via the PI(4,5)P_2_-PLC signaling pathway

**DOI:** 10.3389/fphar.2015.00022

**Published:** 2015-02-11

**Authors:** Masayuki X. Mori, Kyohei Itsuki, Hideharu Hase, Seishiro Sawamura, Tatsuki Kurokawa, Yasuo Mori, Ryuji Inoue

**Affiliations:** ^1^Department of Synthetic and Biological Chemistry, School of Engineering, Kyoto UniversityKyoto, Japan; ^2^Faculty of Dental Science, Kyushu UniversityFukuoka, Japan; ^3^Department of Physiology, School of Medicine, Fukuoka UniversityFukuoka, Japan

**Keywords:** receptor-operated calcium current, TRPC channels, PIP2, voltage-sensing phosphatase, Ca^2+^ signaling, smooth muscle

## Abstract

Transient receptor potential canonical (TRPC) channels are Ca^2+^-permeable, nonselective cation channels that carry receptor-operated Ca^2+^ currents (ROCs) triggered by receptor-induced, phospholipase C (PLC)-catalyzed hydrolysis of phosphatidylinositol 4,5-bisphosphate [PI(4,5)P_2_]. Within the vasculature, TRPC channel ROCs contribute to smooth muscle cell depolarization, vasoconstriction, and vascular remodeling. However, TRPC channel ROCs exhibit a variable response to receptor-stimulation, and the regulatory mechanisms governing TRPC channel activity remain obscure. The variability of ROCs may be explained by their complex regulation by PI(4,5)P_2_ and its metabolites, which differentially affect TRPC channel activity. To resolve the complex regulation of ROCs, the use of voltage-sensing phosphoinositide phosphatases and model simulation have helped to reveal the time-dependent contribution of PI(4,5)P_2_ and the possible role of PI(4,5)P_2_ in the regulation of ROCs. These approaches may provide unprecedented insight into the dynamics of PI(4,5)P_2_ regulation of TRPC channels and the fundamental mechanisms underlying transmembrane ion flow. Within that context, we summarize the regulation of TRPC channels and their coupling to receptor-mediated signaling, as well as the application of voltage-sensing phosphoinositide phosphatases to this research. We also discuss the controversial bidirectional effects of PI(4,5)P_2_ using a model simulation that could explain the complicated effects of PI(4,5)P_2_ on different ROCs.

## INTRODUCTION ~RECEPTOR-OPERATED Ca^2+^ CURRENTS~

Calcium is a ubiquitous and fundamental messenger that triggers numerous downstream cellular events, including hormone secretion, vasoconstriction, and activity-dependent gene expression, to name a few (Berridge, 2012). There are several mechanisms by which Ca^2+^ signals are generated (Parekh and Putney, 2005). These include voltage-dependent Ca^2+^ influx, Ca^2+^ release from intracellular stores, and store-operated Ca^2+^ influx in response to depletion of the Ca^2+^ stores. In this paper, we will focus on phospholipase C (PLC)-driven Ca^2+^ influx, often described as receptor-operated Ca^2+^ currents (ROCs). The Ca^2+^ signal mediated by ROCs differs from that provided by voltage-dependent Ca^2+^ influx (Somlyo and Somlyo, 1994). However, comparatively little is known about the properties of ROCs and their underlying mechanisms. It is known that in various vertebrate cell types ROCs are generated through the breakdown of phosphatidylinositol 4,5-bisphosphate [PI(4,5)P_2_] by PLC upon receptor stimulation and are marked by Ca^2+^/Na^+^ influx (Bolton, 1979, Putney and Tomita, 2012). The role of ROCs has also been studied in the *Drosophila* phototransduction pathway, wherein TRP channels mediate cation currents in response to photoreceptor activation (Hardie, 2011; Montell, 2011).

**FIGURE 1:**
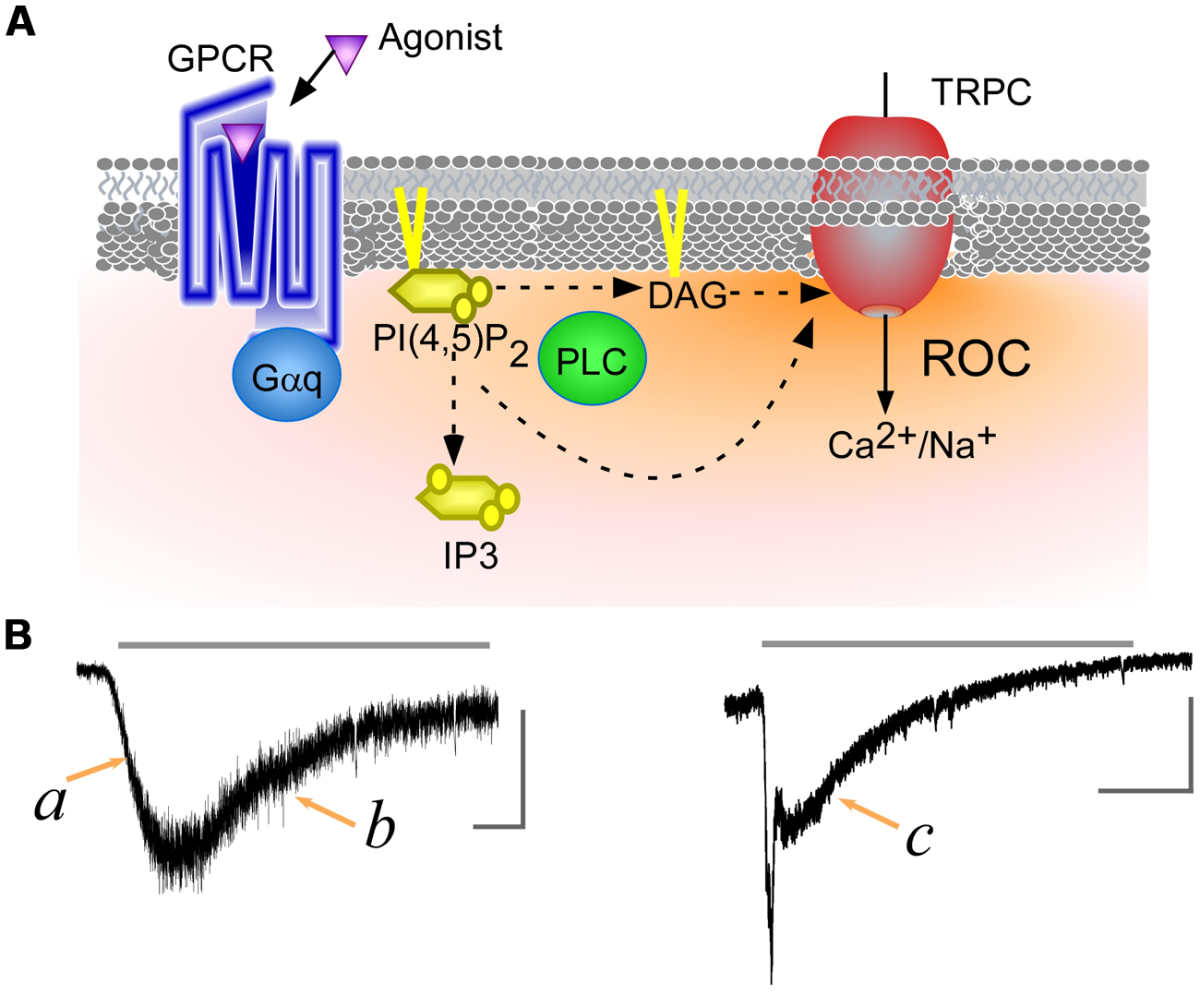
**Transient receptor potential canonical (TRPC) channels receptor-operated Ca^2+^ currents (ROCs). (A)** Schematic representation of ROCs. Binding of an agonist to a G_q_-protein-coupled receptor leads to phospholipase C (PLC) activation. The activated PLC hydrolyzes phosphatidylinositol 4,5-bisphosphate [PI(4,5)P_2_] to produce of diacylglycerol (DAG) and IP_3_. DAG and the reduction of PI(4,5)P_2_ levels directly contribute to TRPC channels activation, while IP_3_ triggers Ca^2+^ release from intracellular stores. **(B)** ROCs through TRPC7 channels. TRPC7 currents were induced using carbachol, a muscarinic receptor agonist (gray line, 100 μM). Left and right panels respectively display currents observed with low and high levels of muscarinic receptor expression (data from Itsuki et al., 2014). The vertical and horizontal gray scale bars indicate 200 pA and 15 s, respectively.

Several TRP channel homologs, known as transient receptor potential canonical (TRPC; canonical), have been cloned from mammalian (Ramsey et al., 2006). Among of these, TRPC2, 3, 6, 7 channels can be activated by diacylglycerol (DAG), a potent lipid messenger produced from PI(4,5)P_2_ by PLC activation (Hofmann et al., 1999). It has been suggested that activation of TRPC4, 5 is PLC-dependent, but with no detectable contribution of DAG (Schaefer et al., 2000). Nevertheless, the linkage between PLC-coupled receptors and TRPC channels is almost universally accepted, and the resulting Ca^2+^ influx is considered to be a ROC (**Figure 1A**).

## PHYSIOLOGICAL PATHWAY FOR ROCs OF TRPC CURRENTS

Transient receptor potential canonical channels are also widely distributed in various other tissues (Beech, 2013). Thus, the upstream of TRPC channels can be diverse, reflecting the physiological context. Within the autonomic nervous system, receptor ligands (e.g., noradrenaline and acetylcholine) are released as transmitters from efferent and afferent sympathetic and parasympathetic nerve fibers at target organs. The effects of noradrenaline are mediated via activation of adrenergic receptors, including the α1A receptor, which is abundantly expressed on venous smooth muscle cells, and has been shown to induce ROCs through TRPC6 channels (Inoue et al., 2001). In cerebellar granule cells, brain-derived neurotrophic factor (BDNF)-induced Ca^2+^ elevation by TRPC channels has been shown to play an essential role in nerve growth cones guidance (Li et al., 2005). Furthermore, pathological contributions of ROCs of TRPC channels have been shown in development of hypertrophy (Onohara et al., 2006; Wu et al., 2010; Seo et al., 2014) and genetic kidney disease ‘focal segmental glomerulosclerosis (FSGS)’ (Mukerji et al., 2007). To emphasize physiological contribution of ROCs, a list of the agonists, receptors, PLC subtypes, and TRPC channels, and their confirmed linkage is presented in the supplementary material (Table S1).

## DYNAMICS OF ROCs

Transient receptor potential canonical channel ROCs can exhibit slow *u*-shaped time dependence or an initial rapid spike followed by a sustained response, depending on the strength of the receptor stimulation (**Figure 1B**). The activation phase of ROCs shows a facilitative or growing curve, irrespective of the agonist concentration applied (**Figure 1B**,a). It has been demonstrated that cytosolic Ca^2+^ potentiates activity of TRPC5 channels (Blair et al., 2009), however, the facilitative responses induced by receptor agonists are less clear to PI(4,5)P_2_. For example, PI(4,5)P_2_ exerts an inhibitory effect on the *Drosophila* TRPC channel, which prompted an intriguing proposal that reductions in the PI(4,5)P_2_ concentration due to PLC hydrolysis may be sufficient to evoke ROCs. In a recent study, however, reducing PI(4,5)P_2_ levels in the absence of PLC activity through rapamycin-induced yeast PI(4,5)P_2_ phosphatase had no effect on TRPL channel activation (Lev et al., 2012).

The decay phase following the peak exhibits an even more curious. Under a low-dose of an agonist application, ROCs gradually disappear without a plateau phase (**Figure 1B**,b). On the other hand, at higher agonist doses ROCs often demonstrate fast inactivation followed by a plateau phase (**Figure 1B**,c). The plateau phase of ROCs also appears in *Drosophila* photoreceptors, where it is known to be dependent on the intensity of the light stimulation. When the light stimulus is very dim, the photoreceptor-operated currents decay to baseline without a clear plateau phase. Brighter stimuli elicit a plateau phase and shortened the decay time from the plateau to baseline (Minke, 1982). In mammalian TRPC channels, such low vs. high doses of receptor agonist application, which would turn out to impact PI(4,5)P_2_ hydrolysis, may evoke this opposing effect, but the pattern is not clearly understood yet. In addition, little is known that receptor’s down-regulation, heteromerization of channel subunits, and their regulatory molecules exert an inhibitory effect on ROCs, and an important goal of future experiments will be to identify and characterize the factors critically involved to generating the ROCs.

## CONTROVERSIAL PI(4,5)P_2_ EFFECTS IN TRP CHANNELS

Phosphatidylinositol 4,5-bisphosphate is located in the inner leaflet of the plasma membrane. In addition to being a substrate for hydrolysis by PLC, PI(4,5)P_2_ plays key roles in the regulation of cytoskeletal organization, cell motility, and a number ion conducting proteins (Balla, 2013). In mammals, 20 of the 28 known TRP channel subtypes are regulated by PI(4,5)P_2_. However, studies of PI(4,5)P_2_ regulation have often reached differing conclusions. For example, in TRPV1, one of the best characterized members of the TRP superfamily, PI(4,5)P_2_ may positively or negatively regulate channel activity (for review, see Rohacs, 2013). In addition, TRPV4 channels, recently further added to the controversy surrounding PI(4,5)P_2_ function. PI(4,5)P_2_ appears to suppress TRPV4 channel activity by binding to an ankyrin domain, while on the other hand PI(4,5)P_2_ can also facilitate TRPV4 channel activity through binding to a *N*-terminal region separate from the ankyrin domain (Garcia-Elias et al., 2013; Takahashi et al., 2014), which implies domain-specific regulation of TRPV4 activation. Furthermore, the effect of PI(4,5)P_2_ on TRPV4 channel activation can depend on the stimulus in the heat or osmo vs chemical (4-α-PPD) stimulations. Under physiological conditions, the breakdown of PI(4,5)P_2_ by PLC is required for activation of TRPC and TRPL channels. Nonetheless, as with the aforementioned TRPV channels, the effect of PI(4,5)P_2_ is controversial. It is noteworthy that the controversial reports, which are well-reviewed (Rohacs, 2013), utilized different cell settings and different approaches to manipulating PI(4,5)P_2_ levels, which raises questions as to whether the PI(4,5)P_2_ manipulations in these studies are comparable (Mori and Inoue, 2014). To overcome that potential limitation, the clear-cut effects of the voltage-sensing phosphatase (VSP) are discussed in the next section.

## VSP AS A MODERN TOOL FOR STUDYING PI(4,5)P_2_ REGULATION

Voltage-sensing phosphatase is a newly standardized tool for studying ion channel regulation by phosphoinositides that provides high temporal resolution and is controllable through the membrane potential. So far, two VSPs from aquatic species have been being applied to ion channel studies. *Ciona intestinalis* (Ci) VSP was the first to be identified as a voltage-sensing phosphoinositide phosphatase (Murata et al., 2005). Identified later was the VSP from *Danio rerio* (Dr), which exhibits only small differences in the substrate specificity from Ci-VSP, mainly in its voltage-sensitivity (Hossain et al., 2008).

**FIGURE 2:**
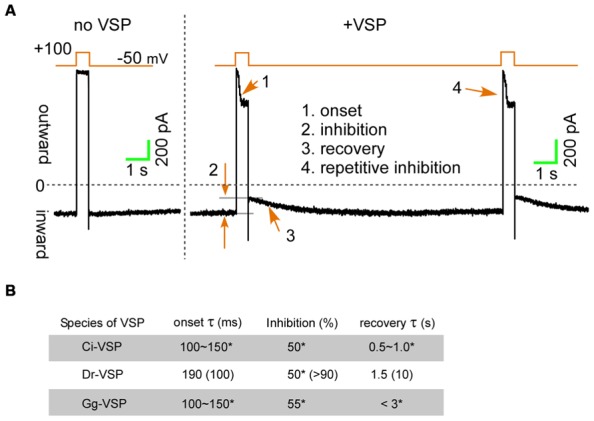
**The inhibition upon the voltage-sensing phosphatases (VSPs) activation. (A)** TRPC6 currents induced by a DAG analog (OAG) are transiently inhibited by Dr-VSP activation (Imai et al., 2012). **(B)** Inhibitory effects of VSPs on TRPC6 and KCNQ2/3 channels. Data for TRPC6 channels are from Imai et al. (2012). Parenthesizes indicate values for KCNQ2/3 channels (Falkenburger et al., 2010). *Unpublished data from experiments in which equal amounts of plasmid harboring cDNA encoding Ci-, Dr-, or Gg-VSP, and TRPC6 were co-transfected into HEK293 cells. Currents were evoked using 50 μM OAG.

Membrane depolarization under voltage-clamp activates VSPs within a few milliseconds, enabling detection of transient effects on ion channels (**Figure 2A**, onset). Hille’s group utilized Dr-VSP, and by measuring onset time course of the current reduction, they estimated the residence time of PI(4,5)P_2_ on the KCNQ2/3 channels to be less than 10 ms, which is more than 400 times faster than the previous estimation (Falkenburger et al., 2010). Because VSPs are only activated during membrane depolarization, upon repolarization of the membrane, PI(4,5)P_2_ levels, and thus channel currents, are able to recover due the presence of endogenous phosphatidylinositol 4-phosphate 5-kinase (PIP5K), which catalyzes position 5 of the inositol ring (Di Paolo and De Camilli, 2006). The time course of the channel current recovery (**Figure 2A**, recovery) is illuminating and reflects the time constant for the functional re-association of PI(4,5)P_2_ with the ion channel. Intriguingly, the recovery constant for the KCNQ2/3 channel and *N*-type Ca^2+^ channel inhibition is more than 10 s, which is much slower than in TRPC3/6/7 channels (1–4 s) or TRPM8 channels (4 s; Falkenburger et al., 2010; Suh et al., 2010; Yudin et al., 2011; Imai et al., 2012). According to the time constants of onset and recovery, higher affinity of PI(4,5)P_2_ for TRPC3 and TRPC6 channels has suggested than that for the KCNQ2/3 or *N*-type Ca^2+^ channels.

To determine the affinity of PI(4,5)P_2_ for ion channels in another voltage clamp study, we controlled the magnitude of PI(4,5)P_2_ reductions by Dr-VSP activation using a step-pulse protocol, which enabled us to determine the affinities of PI(4,5)P_2_ for TRPC3/6/7 channels. The *K*_d_ values obtained ranged from 1 to 10 μM (Itsuki et al., 2014), indicating higher affinity than that of PI(4,5)P_2_ for KCNQ2/3 channels (EC50 ≈ 87 μM; Zhang et al., 2003). This raises the possibility that the affinity of PI(4,5)P_2_ for TRPC channels could be too strong to exert regulatory effects, given the physiological PI(4,5)P_2_ concentration. However, the PI(4,5)P_2_ concentration in cells has been estimated to range from 5 to 10 μM and may vary by around 50% upon activation of signaling (Xu et al., 2003; McLaughlin and Murray, 2005). This concentration range clearly overlaps the dissociation constants for TRPC channels and is consistent with the physiological relevance of PI(4,5)P_2_ regulation. Furthermore, evidence of physical coupling between PLC and TRPC channels has been provided (van Rossum et al., 2005), local PI(4,5)P_2_ depletion may possible to happen nearby TRPC channels.

## COMPARISON OF TRPC6 CHANNELS INHIBITION BETWEEN Ci-, Dr-, AND Gg-VSPs

Voltage-sensing phosphatase In addition to sea ascidian (Ci) and zebra fish (Dr), VSPs have also been identified in chicken (*Gallus gallus*, Gg; Yamaguchi et al., 2014). Among these three VSPs, Dr-VSP appears to be best for manipulating PI(4,5)P_2_, as it is more strongly expressed than Ci-VSP (Hossain et al., 2008) and has higher catalytic specificity toward PI(4,5)P_2_ than Gg-VSP (Yamaguchi et al., 2014). Nonetheless, we compared the effects of Ci-, Dr,- and Gg-VSP on TRPC6 currents. The extent to which the three homologs inhibited TRPC6 currents and the time-courses of their effects differed only with respect to recovery, and were otherwise nearly equal (**Figure 2B**). This finding supports the theory that Ci-, Dr-, and Gg-VSP are all valuable molecular tools for characterizing channel regulation by PI(4,5)P_2_.

## POTENTIAL OF OPPOSING PI(4,5)P_2_ EFFECTS ON A ROC

As described above, reductions in PI(4,5)P_2_ can have opposite effects on TRPC channel activity. We therefore wondered whether this effect could explain the dynamics of both ROCs and the reductions in PI(4,5)P_2_ observed in our recent experiments (Itsuki et al., 2014). So far, no model has incorporated opposing effects of PI(4,5)P_2_ on the regulation of TRPC channel activity. We newly modified to add these effects into a promising model of TRPC6/7 currents, which we called the SPD model (Itsuki et al., 2014). When we then fit simulations generated with the new model to TRPC6 channel ROCs, which were recorded simultaneously with PI(4,5)P_2_ dynamics using a FRET sensor, the simulated ROC was well fitted to the experimentally observed ROC dynamics (Figure S1, upper right panel). Although the simulated PI(4,5)P_2_ dynamics diverged somewhat from the experimental data (Figure S1, lower right panel), we found that bi-directional PI(4,5)P_2_ regulation may be possible if the negative effect site of PI(4,5)P_2_ is a range from 20× less to greater affinity for PI(4,5)P_2_ than the positive effect site for PI(4,5)P_2_ (Table S2).

Phosphatidylinositol 4,5-bisphosphate association sites as well as its physiological importance remains to be addressed in future studies. TRPC channel ROCs show great variation, and the mechanisms, and physiological consequences of that variation are not yet fully understood. Precise detection of signals elicited by receptor stimulation and clear cut evidence of the regulatory factors, including PI(4,5)P_2_, Ca^2+^, will aid our understanding of ROCs within pathophysiological responses.

## Conflict of Interest Statement

The authors declare that the research was conducted in the absence of any commercial or financial relationships that could be construed as a potential conflict of interest.
