# Supplementary material for: Dynamics of receptor-operated Ca2+ currents through TRPC channels controlled via the PI(4,5)P2-PLC signaling pathway
Source: Front Pharmacol. 2015 Feb 11;6:22. doi: 10.3389/fphar.2015.00022 (PMC4324076; doi:10.3389/fphar.2015.00022)
Supplement: Supplementary file 1 [file Data_Sheet_1.PDF]

***Table S1 Reported combinations of agonist-receptor-PLC-TRPC channels***

| Agonist | Receptor | Downstream | Channel | References |
|---|---|---|---|---|
| Angiotensin II | $AT_1$ receptor | PLC | TRPC3,6 | (Saleh et al., 2006) (Onohara et al., 2006) |
| ATP | $P_2Y$ receptor | PLC | TRPC3,7 | (Kamouchi et al., 1999) (Okada et al., 1999) (Inoue et al., 2001) |
| ATP | $P_2Y$ receptor | | TRPC5 | (Okada et al., 1998) |
| Arginine vasopressin | V1a receptor | PLC | TRPC6,7 | (Maruyama et al., 2006) (Itsuki et al., 2014) (Inoue et al., 2001) |
| BDNF | TrkB | PLCγ | TRPC3,6 | (Kamouchi et al., 1999) (Li et al., 1999) (Li et al., 2005) (Jia et al., 2007) (Amaral and Pozzo-Miller, 2007) (Sciarretta et al., 2010) (Vohra et al., 2013) |
| Bradykinin | $B_2R$ ($B_2$ bradykinin receptor) | PLCβ | TRPC3, 6 | (Kamouchi et al., 1999) (Bandyopadhyay et al., 2005) (Leung et al., 2006) |
| Carbachol | muscarinic receptors | | TRPC4,5 | (Lee et al., 2003) (Venkatachalam et al., 2003) (Zeng et al., 2004) (Blair et al., 2009) (Schaefer et al., 2000) (Miller et al., 2011) |
| Carbachol | muscarinic $M_2$, M3 receptor | Gαi/o | TRPC4,5 | (Jeon et al., 2012) (Tsvilovskyy et al., 2009) |
| Carbachol | muscarinic receptor | PLC | TRPC6 | (Monet et al., 2012) (Tsvilovskyy et al., 2009) (Inoue et al., 2001) |
| Cholecystokinin | $CCK_2$ receptor | PLC | TRPC4, 5 | (Grisanti et al., 2012) (Meis et al., 2007) |
| Diacylglycerol (DAG) | | | TRPC2,3,6,7 | (Lucas et al., 2003) (Hofmann et al., 1999) (Okada et al., 1999) |
| Endothelin-1 | ETa receptor | PLC | TRPC3,7 | (Peppiatt-Wildman et al., 2007) |
| EGF | EGF Receptor | PLC | TRPC4 | (Peppiatt-Wildman et al., 2007) (Odell et al., 2005) (Odell et al., 2008) |
| Erythropoietin | Epo-R (Erythropoietin receptor) | PLCγ, IP3R | TRPC3 | (Tong et al., 2008) |
| Histamine | H1 histamine receptor | | TRPC4,5 | (Schaefer et al., 2000) |
| Histamine | H1 histamine receptor | PLC | TRPC3,6 | (Kwon et al., 2007) (Hofmann et al., 1999) |

| | | | | |
|---|---|---|---|---|
| Hypoxia | | DAG accumulation | TRPC6 | (Weissmann et al., 2006) |
| Light | melanopsin | PLCβ4 | TRPC6,7 | (Perez-Leighton et al., 2011) (Xue et al., 2011) |
| Noradrenaline | α-adrenoceptors | PLC | TRPC6 | (Inoue et al., 2001) |
| PDGF | PDGF receptor | PLCγ | TRPC6 | (Jung et al., 2002) |
| 2-heptanone | Vomeronasal receptor | PLC | TRPC2 | (Leypold et al., 2002) |

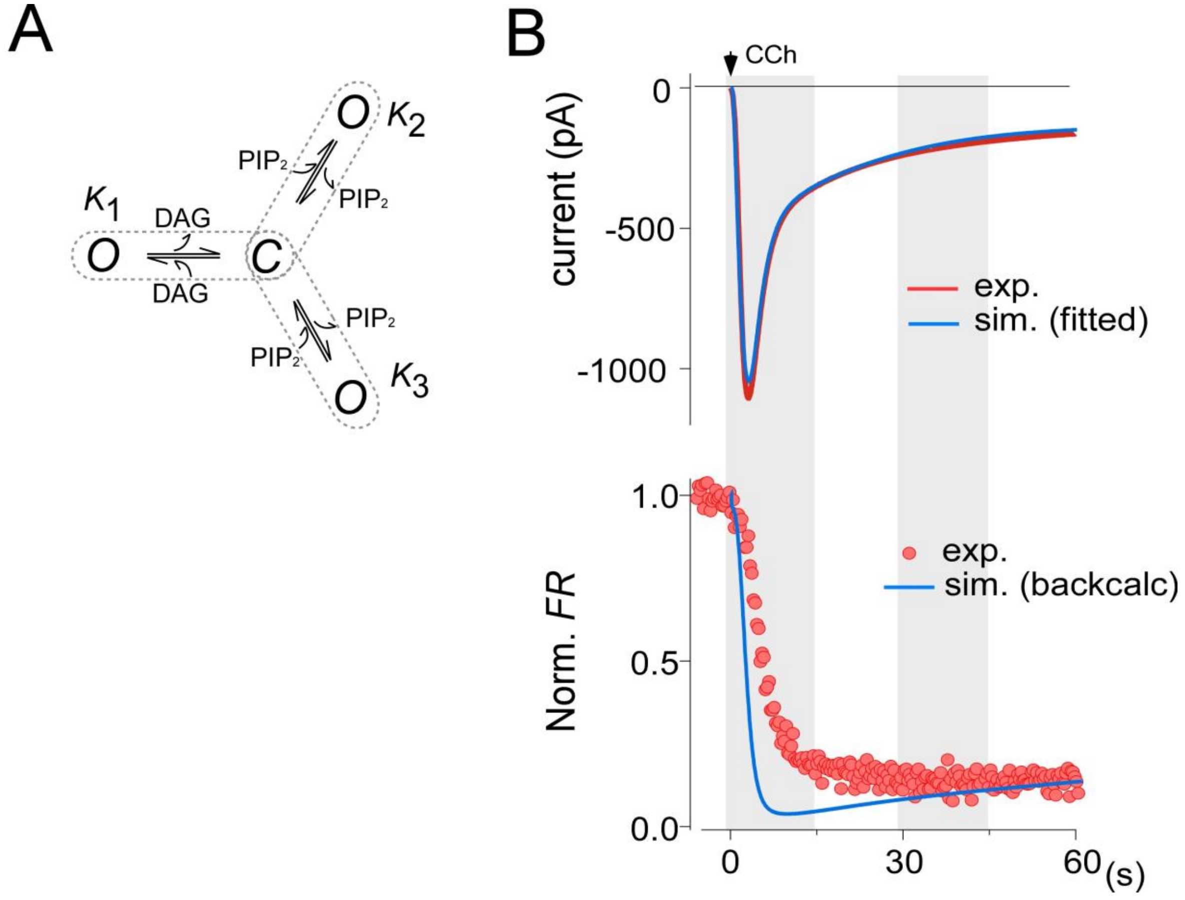


***Figure S1. Fitting to experimentally observed ROCs with an opposite PI(4,5)P$_2$ effect incorporated model.***

(A) PI(4,5)P$_2$ and DAG concentrations are linked to the three-state models. This new model furnished opposite contributions of PI(4,5)P$_2$, in addition to the positive DAG effect. According to this model, the probability of being open ($P$o) versus the concentration of PI(4,5)P$_2$ and DAG relationship can be described as follows,

$$P\mathrm{o} = \left( (K_1 \cdot K_3/(K_2 \cdot \mathrm{d[DAG]})) + (K_1/\mathrm{d[DAG]}) \cdot (K_3/\mathrm{d[PI(4{,}5)P_2]}) + K_3/K_2 + (K_1/\mathrm{d[DAG]}) \cdot (\mathrm{d[PI(4{,}5)P_2]}/K_2) + (K_1/\mathrm{d[DAG]}) + (K_3/\mathrm{d[PI(4{,}5)P_2]}) + (\mathrm{d[PI(4{,}5)P_2]}/K_2) + 1 \right)^{-1},$$

where $K_1$ and $K_2$ or $K_3$ are the equilibrium dissociation constants for DAG and PI(4,5)$P_2$. $K_2$ and $K_3$ are positive and negative PI(4,5)$P_2$ effective sites, respectively. This equation was utilized in our build simulation (Itsuki et al., 2014). (B) Fitting of the receptor-operated TRPC6 current simultaneously measured with PI(4,5)$P_2$ by FRET with this model (top panel, blue line). The experimental data (top panel, red trace) was obtained from the high strength of receptor stimulation carried by CCh-application (100 µM), data obtained from our published data (Itsuki et al., 2014). Back-calculated FRET of PI(4,5)$P_2$ concentrations at the respective points (bottom panel, blue line) was normalized against the time zero FRET strength, and that was overlaid on the experimental FRET signals (bottom panel, red circles).

**Table S2 Resultant parameters by fitting to TRPC6 current with the opposite $PI(4,5)P_2$ regulation incorporated model, shown in Figure S1.**

| Cell | | HEK293 |
|---|---|---|
| Transfected plasmids | | TRPC6/$M_1$R |
| Receptor Agonist (conc. μM) | | Carbachol (100) |
| Description for parameters | | Parameters (unit) |
| 1 | $PI(4,5)P_2$ (μM) at resting | 8.11 |
| 2 | $k_i$_PLC ($s^{-1}$) | 0.74 |
| 3 | $k_{ii}$_DAG kinase ($s^{-1}$) | 0.13 |
| 4 | $k_{iii}$_PA to PIP reactions, ($s^{-1}$) | 0.006 |
| 5 | $k_{iv}$_PIP5K ($s^{-1}$) | 0.030 |
| 6 | $k_v$_$IP_3$ phosphatase ($s^{-1}$) | 0.98 |
| 7 | $\tau_{rd}$ (s) | 2.19 |
| 8 | $\tau_{sd}$ (s) | 11.5 |
| 9 | Rd_f (no unit) | 0.6 |
| 10 | Sd_f (no unit) | 0.4 |
| 11 | spot (distance global to local (μm)) | 0.88 |
| 12 | dcoef of $PI(4,5)P_2$ ($\mu m^2/s$) | 0.95 |
| 13 | Ratio of local $k_i$ / global $k_i$ | 0.81 |
| 14 | Activation delay factor | 0.011 |
| 15 | Activation power factor | 0.25 |
| 16 | PI(4)P (μM) | 11.70 |
| 17 | The number of channels | 6278 |
| 18 | $K_1$ ($k_d$ for DAG1, μM) | 20.00 |
| 19 | $K_2$ ($k_d$ for $PI(4,5)P_2$, μM) (positive) | 1.66 |
| 20 | $K_3$ ($k_d$ for $PI(4,5)P_2$, μM) (negative) | 28.60 |

## *REFERENCES*


Amaral, M.D., and Pozzo-Miller, L. (2007). TRPC3 channels are necessary for brain-derived neurotrophic factor to activate a nonselective cationic current and to induce dendritic spine formation. *J Neurosci* 27, 5179-5189.

Bandyopadhyay, B.C., Swaim, W.D., Liu, X., Redman, R.S., Patterson, R.L., and Ambudkar, I.S. (2005). Apical localization of a functional TRPC3/TRPC6-Ca2+-signaling complex in polarized epithelial cells. Role in apical Ca2+ influx. *J Biol Chem* 280, 12908-12916.

Blair, N.T., Kaczmarek, J.S., and Clapham, D.E. (2009). Intracellular calcium strongly potentiates agonist-activated TRPC5 channels. *J Gen Physiol* 133, 525-546.

Grisanti, L.A., Kurada, L., Cilz, N.I., Porter, J.E., and Lei, S. (2012). Phospholipase C not protein kinase C is required for the activation of TRPC5 channels by cholecystokinin. *Eur J Pharmacol* 689, 17-24.

Hofmann, T., Obukhov, A.G., Schaefer, M., Harteneck, C., Gudermann, T., and Schultz, G. (1999). Direct activation of human TRPC6 and TRPC3 channels by diacylglycerol. *Nature* 397, 259-263.

Inoue, R., Okada, T., Onoue, H., Hara, Y., Shimizu, S., Naitoh, S., Ito, Y., and Mori, Y. (2001). The transient receptor potential protein homologue TRP6 is the essential component of vascular alpha(1)-adrenoceptor-activated Ca(2+)-permeable cation channel. *Circ Res* 88, 325-332.

Itsuki, K., Imai, Y., Hase, H., Okamura, Y., Inoue, R., and Mori, M.X. (2014). PLC-mediated PI(4,5)P2 hydrolysis regulates activation and inactivation of TRPC6/7 channels. *J Gen Physiol* 143, 183-201.

Jeon, J.P., Hong, C., Park, E.J., Jeon, J.H., Cho, N.H., Kim, I.G., Choe, H., Muallem, S., Kim, H.J., and So, I. (2012). Selective Galphai subunits as novel direct activators of transient receptor potential canonical (TRPC)4 and TRPC5 channels. *J Biol Chem* 287, 17029-17039.

Jia, Y., Zhou, J., Tai, Y., and Wang, Y. (2007). TRPC channels promote cerebellar granule neuron survival. *Nat Neurosci* 10, 559-567.

Jung, S., Strotmann, R., Schultz, G., and Plant, T.D. (2002). TRPC6 is a candidate channel involved in receptor-stimulated cation currents in A7r5 smooth muscle cells. *Am J Physiol Cell Physiol* 282, C347-359.

Kamouchi, M., Philipp, S., Flockerzi, V., Wissenbach, U., Mamin, A., Raeymaekers, L., Eggermont, J., Droogmans, G., and Nilius, B. (1999). Properties of heterologously expressed hTRP3 channels in bovine pulmonary artery endothelial cells. *J Physiol* 518 Pt 2, 345-358.

Kwon, Y., Hofmann, T., and Montell, C. (2007). Integration of phosphoinositide- and calmodulin-mediated regulation of TRPC6. *Mol Cell* 25, 491-503.

Lee, Y.M., Kim, B.J., Kim, H.J., Yang, D.K., Zhu, M.H., Lee, K.P., So, I., and Kim, K.W. (2003). TRPC5 as a candidate for the nonselective cation channel activated by muscarinic stimulation in murine stomach. *Am J Physiol Gastrointest Liver Physiol* 284, G604-616.

Leung, P.C., Cheng, K.T., Liu, C., Cheung, W.T., Kwan, H.Y., Lau, K.L., Huang, Y., and Yao, X. (2006). Mechanism of non-capacitative Ca2+ influx in response to bradykinin in vascular endothelial cells. *J Vasc Res* 43, 367-376.

Leypold, B.G., Yu, C.R., Leinders-Zufall, T., Kim, M.M., Zufall, F., and Axel, R. (2002). Altered sexual and social behaviors in trp2 mutant mice. *Proc Natl Acad Sci U S A* 99, 6376-6381.

Li, H.S., Xu, X.Z., and Montell, C. (1999). Activation of a TRPC3-dependent cation current through the neurotrophin BDNF. *Neuron* 24, 261-273.

Li, Y., Jia, Y.C., Cui, K., Li, N., Zheng, Z.Y., Wang, Y.Z., and Yuan, X.B. (2005). Essential role of TRPC channels in the guidance of nerve growth cones by brain-derived neurotrophic factor. *Nature* 434, 894-898.

Lucas, P., Ukhanov, K., Leinders-Zufall, T., and Zufall, F. (2003). A diacylglycerol-gated cation channel in vomeronasal neuron dendrites is impaired in TRPC2 mutant mice: mechanism of pheromone transduction. *Neuron* 40, 551-561.

Maruyama, Y., Nakanishi, Y., Walsh, E.J., Wilson, D.P., Welsh, D.G., and Cole, W.C. (2006). Heteromultimeric TRPC6-TRPC7 channels contribute to arginine vasopressin-induced cation current of A7r5 vascular smooth muscle cells. *Circ Res* 98, 1520-1527.

Meis, S., Munsch, T., Sosulina, L., and Pape, H.C. (2007). Postsynaptic mechanisms underlying responsiveness of amygdaloid neurons to cholecystokinin are mediated by a transient receptor potential-like current. *Mol Cell Neurosci* 35, 356-367.

Miller, M., Shi, J., Zhu, Y., Kustov, M., Tian, J.B., Stevens, A., Wu, M., Xu, J., Long, S., Yang, P., Zholos, A.V., Salovich, J.M., Weaver, C.D., Hopkins, C.R., Lindsley, C.W., Mcmanus, O., Li, M., and Zhu, M.X. (2011). Identification of ML204, a novel potent antagonist that selectively modulates native TRPC4/C5 ion channels. *J Biol Chem* 286, 33436-33446.

Monet, M., Francoeur, N., and Boulay, G. (2012). Involvement of phosphoinositide 3-kinase and PTEN protein in mechanism of activation of TRPC6 protein in vascular smooth muscle cells. *J Biol Chem* 287, 17672-17681.

Odell, A.F., Scott, J.L., and Van Helden, D.F. (2005). Epidermal growth factor induces tyrosine phosphorylation, membrane insertion, and activation of transient receptor potential channel 4. *J Biol Chem* 280, 37974-37987.

Odell, A.F., Van Helden, D.F., and Scott, J.L. (2008). The spectrin cytoskeleton influences the surface expression and activation of human transient receptor potential channel 4 channels. *J Biol Chem* 283, 4395-4407.

Okada, T., Inoue, R., Yamazaki, K., Maeda, A., Kurosaki, T., Yamakuni, T., Tanaka, I., Shimizu, S., Ikenaka, K., Imoto, K., and Mori, Y. (1999). Molecular and functional characterization of a novel mouse transient receptor potential protein homologue TRP7. Ca(2+)-permeable cation channel that is constitutively activated and enhanced by stimulation of G protein-coupled receptor. *J Biol Chem* 274, 27359-27370.

Okada, T., Shimizu, S., Wakamori, M., Maeda, A., Kurosaki, T., Takada, N., Imoto, K., and Mori, Y. (1998). Molecular cloning and functional characterization of a novel receptor-activated TRP Ca2+ channel from mouse brain. *J Biol Chem* 273, 10279-10287.

Onohara, N., Nishida, M., Inoue, R., Kobayashi, H., Sumimoto, H., Sato, Y., Mori, Y., Nagao, T., and Kurose, H. (2006). TRPC3 and TRPC6 are essential for angiotensin II-induced cardiac hypertrophy. *Embo J* 25, 5305-5316.

Peppiatt-Wildman, C.M., Albert, A.P., Saleh, S.N., and Large, W.A. (2007). Endothelin-1 activates a Ca2+-permeable cation channel with TRPC3 and TRPC7 properties in rabbit coronary artery myocytes. *J Physiol* 580, 755-764.

Perez-Leighton, C.E., Schmidt, T.M., Abramowitz, J., Birnbaumer, L., and Kofuji, P. (2011). Intrinsic phototransduction persists in melanopsin-expressing ganglion cells lacking diacylglycerol-sensitive TRPC subunits. *Eur J Neurosci* 33, 856-867.

Saleh, S.N., Albert, A.P., Peppiatt, C.M., and Large, W.A. (2006). Angiotensin II activates two cation conductances with distinct TRPC1 and TRPC6 channel properties in rabbit mesenteric artery myocytes. *J Physiol* 577, 479-495.

Schaefer, M., Plant, T.D., Obukhov, A.G., Hofmann, T., Gudermann, T., and Schultz, G. (2000). Receptor-mediated regulation of the nonselective cation channels TRPC4 and TRPC5. *J Biol Chem* 275, 17517-17526.

Sciarretta, C., Fritzsch, B., Beisel, K., Rocha-Sanchez, S.M., Buniello, A., Horn, J.M., and Minichiello, L. (2010). PLCgamma-activated signalling is essential for TrkB mediated sensory neuron structural plasticity. *BMC Dev Biol* 10, 103.

Tong, Q., Hirschler-Laszkiewicz, I., Zhang, W., Conrad, K., Neagley, D.W., Barber, D.L., Cheung, J.Y., and Miller, B.A. (2008). TRPC3 is the erythropoietin-regulated calcium channel in human erythroid cells. *J Biol Chem* 283, 10385-10395.

Tsvilovskyy, V.V., Zholos, A.V., Aberle, T., Philipp, S.E., Dietrich, A., Zhu, M.X., Birnbaumer, L., Freichel, M., and Flockerzi, V. (2009). Deletion of TRPC4 and TRPC6 in mice impairs smooth muscle contraction and intestinal motility in vivo. *Gastroenterology* 137, 1415-1424.

Venkatachalam, K., Zheng, F., and Gill, D.L. (2003). Regulation of canonical transient receptor potential (TRPC) channel function by diacylglycerol and protein kinase C. *J Biol Chem* 278, 29031-29040.

Vohra, P.K., Thompson, M.A., Sathish, V., Kiel, A., Jerde, C., Pabelick, C.M., Singh, B.B., and Prakash, Y.S. (2013). TRPC3 regulates release of brain-derived neurotrophic factor from human airway smooth muscle. *Biochim Biophys Acta* 1833, 2953-2960.

Weissmann, N., Dietrich, A., Fuchs, B., Kalwa, H., Ay, M., Dumitrascu, R., Olschewski, A., Storch, U., Mederos Y Schnitzler, M., Ghofrani, H.A., Schermuly, R.T., Pinkenburg, O., Seeger, W., Grimminger, F., and Gudermann, T. (2006). Classical transient receptor potential channel 6 (TRPC6) is essential for hypoxic pulmonary vasoconstriction and alveolar gas exchange. *Proc Natl Acad Sci U S A* 103, 19093-19098.

Xue, T., Do, M.T., Riccio, A., Jiang, Z., Hsieh, J., Wang, H.C., Merbs, S.L., Welsbie, D.S., Yoshioka, T., Weissgerber, P., Stolz, S., Flockerzi, V., Freichel, M., Simon, M.I., Clapham, D.E., Yau, K.W. (2011) Melanopsin signalling in mammalian iris and retina. *Nature*. 479 67-73.

Zeng, F., Xu, S.Z., Jackson, P.K., McHugh, D., Kumar, B., Fountain, S.J., and Beech, D.J. (2004) Human TRPC5 channel activated by a multiplicity of signals in a single cell. *J Physiol*. 559, 739-750.
